# Bone marrow mesenchymal stem cells overexpressing hepatocyte growth factor ameliorate hypoxic–ischemic brain damage in neonatal rats

**DOI:** 10.1515/tnsci-2020-0204

**Published:** 2021-12-17

**Authors:** Wen Zeng, Yu Wang, Yufeng Xi, Guoqing Wei, Rong Ju

**Affiliations:** Department of Neonatology, Chengdu Women’s and Children’s Central Hospital, School of Medicine, University of Electronic Science and Technology of China, Chengdu, 611731, Sichuan, China

**Keywords:** hypoxic–ischemic brain damage, bone marrow mesenchymal stem cell, hepatocyte growth factor, adenoviral vector, extracellular signal-regulated kinase

## Abstract

**Objectives:**

Hypoxic–ischemic brain damage (HIBD) is a major cause of brain injury in neonates. Bone marrow mesenchymal stem cells (BMSCs) show therapeutic potential for HIBD, and genetic modification may enhance their neuroprotective effects. The goal of this study was to investigate the neuroprotective effects of hepatocyte growth factor (HGF)-overexpressing BMSCs (BMSCs-HGF) against HIBD and their underlying mechanisms.

**Methods:**

BMSCs were transfected with HGF using adenoviral vectors. HIBD models were established and then BMSCs were transplanted into the brains of HIBD rats via intraventricular injection. 2,3,5-Triphenyltetrazolium chloride (TTC) staining was used to measure cerebral infarction volumes. *In vitro*, primary cultured cortical neurons were co-cultured with BMSCs in a Transwell plate system. Oxygen–glucose deprivation (OGD) was applied to imitate hypoxic–ischemic insult, and PD98059 was added to the culture medium to block the phosphorylation of extracellular signal-regulated kinase (ERK). Cell apoptosis was determined using TUNEL staining. The expression of HGF was measured by immunofluorescence, real-time quantitative PCR (RT-qPCR), and western blots. The expression of phosphorylated ERK (p-ERK) and B-cell lymphoma-2 (Bcl-2) was measured by western blots.

**Results:**

HGF-gene transfection promoted BMSC proliferation. Moreover, BMSCs-HGF decreased HIBD-induced cerebral infarction volumes and enhanced the protective effects of the BMSCs against HIBD. BMSCs-HGF also increased expression of HGF, p-ERK, and Bcl-2 in brain tissues. *In vitro*, BMSC-HGF protected neurons against OGD-induced apoptosis. Inhibition of ERK phosphorylation abolished the neuroprotective effect of BMSCs-HGF against OGD.

**Conclusions:**

BMSCs-HGF is a potential treatment for HIBD and that the ERK/Bcl-2 pathway is involved in the underlying neuroprotective mechanism.

## 1 Introduction

Neonatal hypoxic–ischemic brain damage (HIBD) can be detrimental to the neonatal central nervous system both in full-term infants and in preterm infants [1]. To date, many neuroprotective strategies have been proposed, but only therapeutic hypothermia has been approved to treat moderate or severe HIBD [2]. Moreover, neonates with mild HIBD or with a gestational age <35 weeks, who are still at risk of brain injury, do not meet the current criteria for therapeutic hypothermia [3]. Additionally, therapeutic hypothermia is only partially neuroprotective in some severe cases [4]. Therefore, further research is still required to explore more feasible and effective treatment strategies for HIBD.

As known, HIBD is mainly caused by perinatal asphyxia, which would reduce cerebral blood flow, disrupt the delivery of oxygen and glucose to the brain, and lead to anaerobic metabolism. This leads to a cascade of reactions, such as adenosine triphosphate depletion, glutamate and free radicals release, ion pump, and mitochondrial dysfunction [5,6]. Eventually, neurons are subjected to apoptosis and necrosis, leading to neurological deficits. Therefore, reducing neuronal death and promoting neuronal survival could be a promising strategy for improving HIBD sequelae.

Stem cell therapy is emerging as a promising therapeutic approach for various diseases, including HIBD, due to their abilities to self-renew and differentiate [7]. Mesenchymal stem cells (MSCs) are a subset of multipotent stem cells that are present in both adult and neonatal tissues and fluids, such as bone marrow, placenta, adipose tissue, umbilical cord tissue, and umbilical cord blood. Bone marrow mesenchymal stem cells (BMSCs) are among the most commonly used stem cells for therapeutic purposes, because they are easily isolated and exhibit a high survival rate after transplantation due to their relatively low immunogenicity [8,9]. Previous studies have indicated that BMSC-based treatments have protective effects against HI injury [10,11,12,13]. There is evidence that BMSCs can be induced to differentiate into neurons and migrate to damaged regions [14,15]. Moreover, their ability to activate endogenous neural stem cells, promote local angiogenesis and vascular remodeling, modulate immunity, and secrete trophic factors may further contribute to their protective potential in brain injury [16,17,18,19,20]. However, the death of BMSCs after transplantation is a major limitation of cell-based therapies. During HI insults, the hypoxic and glucose-deficient environment may have detrimental effects on BMSCs after transplantation [21]. Genetical modification of BMSCs appears to be a suitable strategy to enhance cell survival and improve their therapeutic applicability.

Hepatocyte growth factor (HGF) is a potent pleiotropic cytokine with multiple functions, including promoting mitogenesis, morphogenesis, angiogenesis, and anti-apoptosis, so HGF is highly involved in tissue regeneration [22]. By binding to its specific receptor, c-Met, HGF activates several downstream signaling pathways, including the extracellular signal-regulated kinase (ERK) pathway, and modulates cell proliferation, apoptosis, migration, and angiogenesis of endothelial cells. Therefore, HGF plays a neuroprotective role by regulating neurogenesis, anti-apoptosis, and angiogenesis [23,24], with the potential to be a candidate gene for BMSC modification.

To elucidate the neuroprotective effects of HGF-overexpressing BMSCs on HIBD, a neonatal HIBD rat model was used. In the present study, BMSCs genetically engineered to overexpress HGF were injected into the brains of HIBD rats, in order to investigate the neuroprotective effects and possible mechanisms of HGF-overexpressing BMSCs on HIBD. Furthermore, the underlying mechanisms were verified in primary cortical neurons, which were co-cultured with BMSCs in a Transwell plate system.

## 2 Materials and methods

### 2.1 Isolation and culture of rat BMSCs

BMSCs were harvested from 4 week-old male Sprague–Dawley (SD) rats (*n* = 4) as previously described [25]. In brief, the bone marrow was rinsed out from hind limbs using Dulbecco’s modified Eagle’s medium (DMEM; Thermo Fisher Scientific, Waltham, MA, USA). Then, the bone marrow filtrate was collected and centrifuged (400×*g* for 3 min). Cells were suspended in DMEM containing 10% fetal bovine serum (FBS; Thermo Fisher Scientific) and incubated at 37°C with 5% CO_2_ for 24 h (1 × 10^4^ cells per well), and nonadherent cells were removed by rinsing with phosphate-buffered saline (PBS). Then, fresh medium was added and replaced every 3 days. After 7 days of growth, the adherent cells become confluent, and this stage was designated as primary passage P0. Further, P0 cells were passaged at a ratio of 1:2. BMSCs at passage P3 were harvested and used for further experiments.

### 2.2 Flow cytometry

BMSCs were characterized by flow cytometry analysis. Cultured cells were trypsinized and resuspended in PBS containing 1% bovine serum albumin (Thermo Fisher Scientific). Cell suspensions were incubated with Alexa Fluor 647-conjugated antibodies against a cluster of differentiation (CD)29, CD90, and CD45 (1:100, all from Santa Cruz Biotechnology, Dallas, TX, USA), respectively, at room temperature (RT) for 30 min in the dark. Isotype-matched IgG served as negative controls. Labeled cells were resuspended in PBS and analyzed by flow cytometry (BD Biosciences, Franklin Lakes, NJ, USA).

### 2.3 Adenoviral vectors and gene transfection of BMSCs

The recombinant adenovirus carrying rat HGF gene, pDC316-mCMV-EGFP-HGF (Ad-HGF), and control adenovirus with the reporter gene of green fluorescent protein (GFP), pDC316-mCMV-EGFP (Ad-GFP), were purchased from Western Biomedical Technology (Chongqing, China). Ad-HGF and Ad-GFP were amplified in 293A cells. P3 BMSCs were infected with the recombinant adenovirus by incubation for 24 h at a multiplicity of infection (MOI) of 150. BMSCs infected with Ad-HGF were defined as BMSCs-HGF, while BMSCs infected with Ad-GFP were termed BMSCs-GFP and represented the negative control.

### 2.4 Cell counting Kit-8 (CCK-8) assay

Cell proliferation was evaluated using the CCK-8 (Dojindo, Osaka, Japan) following the manufacturer’s instructions. BMSCs were seeded onto the 96-well plates (5 × 10^4^ cells per well) and 10 μL of the CCK-8 reagent was added to each well. The optical density (OD) at a wavelength of 450 nm was measured using a microplate reader (Bio-Rad, Hercules, CA, USA). CCK-8 assays were performed once daily for 5 days after transfection to determine the proliferation of BMSCs.

### 2.5 Enzyme-linked immunosorbent assay (ELISA)

The protein level of HGF in the cultured BMSC, BMSC-GFP, and BMSC-HGF supernatant was assessed using an ELISA Kit (R&D Systems, Minneapolis, MN, USA), performed following the manufacturer’s protocols at 48 h after transfection with adenoviral vectors. Absorbance at 450 nm was measured with a microplate reader (Bio-Rad).

### 2.6 Establishment of HIBD rat model and BMSC transplantation

The HIBD rat model was established from 7 day-old SD rats (total number of rats was 60, *n* = 15 per experimental group) as previously described [26,27]. Rat pups were anesthetized by 3% isoflurane inhalation and the right carotid arteries were ligated. After a 2 h recovery from anesthesia and surgical procedure, the pups were placed in a hypoxia chamber under 8% O_2_ and 92% N_2_ for a 2.5 h hypoxic exposure. After the hypoxic–ischemic (HI) insult, pups were randomly divided into an HI group, a BMSC-HGF group, and a BMSC-GFP group. Under stereotactic guidance, rats in the BMSC-HGF group and the BMSC-GFP group received slowly right lateral ventricular injection of BMSC-HGF and BMSC-GFP, respectively (1 × 10^6^ cells in 3 μL of DMEM). The HI group received a right lateral ventricular injection of 3 μL of DMEM without cells. Rat pups in the Sham group only were subjected to exposure to the right carotid artery and right lateral ventricular injection of 3 μL of DMEM without cells and hypoxia. The lateral ventricular injection was performed under stereotactic guidance, and the injection coordinates were as follows: 0.5 mm caudal to bregma, 2 mm right of bregma, and 2.5 mm in depth.

### 2.7 Immunofluorescence staining

At 24 h after BMSCs-HGF or BMSCs-GFP transplantation, rats were deeply anesthetized with 3% isoflurane and transcardially perfused with 4% paraformaldehyde. Then, brains (*n* = 3 per experimental group) from different experimental groups were harvested, subjected to gradient hydration with 15, 20, and 30% sucrose solutions, and embedded in OCT. Next, 10 μm brain slices were sectioned coronally for immunofluorescence. The sections were incubated with primary antibody anti-HGF (1:200; Santa Cruz Biotechnology) at 4°C overnight. After being rinsed with PBS 3 times, the sections were incubated with secondary IgG Alexa Fluor 647 antibody (1:800; Abcam, Cambridge, MA, USA) at 37°C for 1 h. The cell nuclei of brain slices were stained with 4′,6-diamidino-2-phenylindole, dihydrochloride (DAPI, Beyotime, Shanghai, China) at RT for 15 min. All images were captured using a fluorescence microscope (Nikon, Tokyo, Japan).

### 2.8 2,3,5-Triphenyltetrazolium chloride (TTC) staining

The cerebral infarct volume was assessed using TTC staining at 72 h post-BMSC transplantation. Brains (*n* = 4 per experimental group) were cut into 2 mm coronal slices and incubated in a 1% TTC solution (Sigma-Aldrich, St. Louis, MO, USA) at 37°C for 30 min. White- and red-stained areas indicated ischemic and nonischemic tissues, respectively. The relative cerebral infarct volume in the brain tissue was analyzed using ImageJ software (NIH).

### 2.9 Primary cultures of rat cortical neurons

Primary cortical neurons were prepared as previously described [28]. The cortical tissue was obtained from the brains of SD rat embryos (E15–16), then minced into small pieces. After being digested with 0.125% trypsin (Beyotime) at 37°C for 20 min, the cell suspension was centrifuged (400×*g* for 3 min) at 4°C. Cells were suspended in a DMEM supplement containing 10% FBS and plated on plates coated with 0.1 mg/mL poly-d-lysine (all obtained from Thermo Fisher Scientific). The cultures were incubated with 5% CO_2_ at 37°C for 4 h and then the DMEM supplement was replaced with a neurobasal medium with 2% B27 supplements, 2 mM glutaMAX, and 100 U/mL penicillin/streptomycin (all obtained from Thermo Fisher Scientific). Half of the medium was replaced every 3 days.

### 2.10 Transwell co-culture and oxygen–glucose deprivation (OGD)

Cultured neurons were used on days 7–10 for following experiments, which were randomly divided into five groups: (i) control; (ii) OGD; (iii) OGD + BMSCs-GFP; (iv) OGD + BMSCs-HGF; and (v) OGD + BMSCs-HGF + PD98059.

Neurons in the control group were cultured normally, without OGD or co-culture with BMSCs. Neurons in the OGD group were subjected to OGD solely, without co-culture with BMSCs. Neurons in OGD + BMSCs-GFP and OGD + BMSCs-HGF were co-cultured with BMSCs-GFP and BMSCs-HGF, respectively, and then subjected to OGD. Neurons in OGD + BMSCs-HGF + PD98059 were co-cultured with BMSCs-HGF and then subjected to OGD with 20 µM PD98059 (Sigma-Aldrich), the inhibitor of the extracellular signal-regulated kinase (ERK)-activating kinase.

Co-cultures of neurons and BMSCs were grown in a 24-well Transwell plate system (0.4 μm pore size; Millipore, Billerica, MA, USA). Briefly, neurons (2 × 10^5^) were seeded in the lower compartment of a 24-well Transwell system, while BMSCs (1 × 10^5^) were cultured in the insert according to the experimental group. After 24 h co-culture, neurons were subjected to OGD.

OGD was performed to initiate the HI insult *in vitro*. Neurons were washed with glucose-free medium (125 mM NaCl, 24 mM NaHCO_3_, 2.8 mM KCl, 2 mM CaCl_2_, 2 mM HEPES, 1.5 mM MgCl_2_, 0.83 mM NaH_2_PO_4_, 0.05 mM MgSO_4_) 3 times, and then placed in an anaerobic incubator containing 95% N_2_ and 5% CO_2_ at 37°C for 4 h in the same glucose-free medium. PD98059 (20 µM, Sigma-Aldrich) was added in the medium of the OGD + BMSCs-HGF + PD98059 group. After OGD, neurons were incubated in a normal culture medium with 5% CO_2_ for 4 h at 37°C and then collected for follow-up experiments.

### 2.11 Western blotting

Protein was extracted from total brains (*n* = 4 per experimental group, at 72 h after the transplantation of BMSCs) and cultured cortical neurons (at 4 h after OGD insult). A 50 µg protein aliquot was subjected to 10% sodium dodecyl sulfate-polyacrylamide gel electrophoresis gels and blotted onto polyvinylidene difluoride membrane (Millipore). After blocking with 5% fat-free milk at RT for 2 h, blots were incubated with primary antibodies at 4°C overnight. The following primary antibodies were used: anti-HGF (1:500), anti-p-ERK (1:500), anti-B-cell lymphoma-2 (Bcl-2, 1:500), and anti-β-actin (1:500) (all obtained from Santa Cruz Biotechnology). After washing, the blots were incubated with secondary antibodies (1:200, Santa Cruz Biotechnology) at RT for 2 h. Finally, membranes were washed and specific bands were detected with an enhanced chemiluminescence system (ECL, Pierce, Rockford, IL, USA). Data from protein densitometry were quantitatively analyzed using Labworks Analysis Software (Upland, CA, USA) and normalized to levels of the housekeeping protein, β-actin.

### 2.12 Real-time quantitative polymerase chain reaction (RT-qPCR)

Total RNA was extracted from BMSCs (at 48 h after transfection with adenoviral vectors) and brains (*n* = 4 per experimental group, at 72 h after the transplantation of BMSCs), respectively, using TRIzol (Thermo Fisher Scientific). cDNA was obtained using the Takara Reverse Transcription Kit (Takara Bio, Kyoto, Japan) following the manufacturer’s protocols. RT-qPCR was conducted using SYBR Green PCR Master Mix (Bio-Rad). The total volume of the mixture was 50 µL, and the RT-qPCR conditions were: 95°C for 4 min, followed by 35 × 3-temperature cycles (95°C for 20 s, 60°C for 30 s, and 72°C for 30 s). β-Actin was used as the internal control. Relative expression was calculated using the 2^−ΔΔCq^ method. The following specific primer pairs were used in this study:

(i) HGF: sense, 5′-CGCAAATGGGCGGTAGCGTG-3′;

antisense, 5′-GCGAATCCCAACGCTGACACGGA-3′;

(ii) β-Actin: sense, 5′-CCCATCTATGAGGGTTACGC-3′;

antisense, 5′-TTTAATGTCACGCACGATTTC-3′.

### 2.13 Terminal deoxyribonucleotidyl transferase-mediated dUTP–digoxigenin nick-end labeling (TUNEL) staining

TUNEL staining assay (Roche, Basel, Switzerland) was applied to determine apoptotic neurons. Briefly, following fixation in 4% paraformaldehyde at RT for 20 min and incubation in a permeabilization solution (0.1% Triton X-100 in 0.1% sodium citrate) at 4°C for 2 min, slices were incubated with the TUNEL reaction mixture reagent at 37°C for 1 h. Subsequently, the slices were stained with DAPI at RT for 5 min. A number of TUNEL positive cells and the total number of cells in five adjacent visual fields in one slice were counted, and the percentage of TUNEL positive cells was calculated. Four different cultures of each group were counted.

### 2.14 Statistical analysis

SPSS 22.0 software was used to analyze the experimental data. All data were expressed as the mean ± standard deviation (SD). Statistical differences between multiple groups were analyzed using one-way analysis of variance (ANOVA) followed by the least significant difference (LSD) *t*-test. *P* < 0.05 was considered as the threshold for statistical significance.


**Ethical approval:** The research related to animals’ use has complied with all the relevant national regulations and institutional policies for the care and use of animals. All animal procedures were in compliance with the National Institutes of Health (NIH) Guidelines for the Care and Use of Laboratory Animals and with local laws. This study was approved by the Ethics Committee of Chengdu Women’s and Children’s Central Hospital.

## 3 Results

### 3.1 Culture and characterization of BMSCs

**Figure 1:**
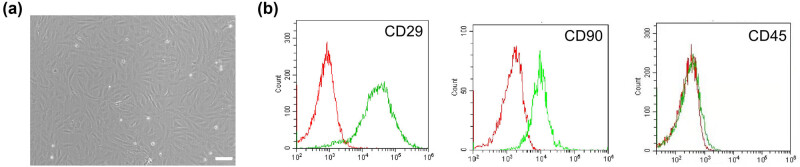
Culture and identification of BMSCs. (a) Morphology of BMSCs after 7 days of culture under a light microscope. Scale bar = 10 μm. (b) Representative flow cytometry plots of CD29, CD90, and CD45 levels in cultured BMSCs. Red and green curves represent the isotype controls and markers, respectively. Flow cytometry showed the positive CD29 and CD90 expression and the negligible CD45 expression.

BMSCs were isolated and cultured from the bone marrow of rats, exhibiting a spindle-shaped or partly spiral-like morphology after 7 days of culture (Figure 1a). Cultured BMSCs were characterized by flow cytometry as positive for CD29 and CD90, and the negligible CD45 expression, thus confirming their identity (Figure 1b).

### 3.2 BMSCs-HGF show enhanced HGF expression and proliferation compared to nontransfected BMSCs

**Figure 2:**
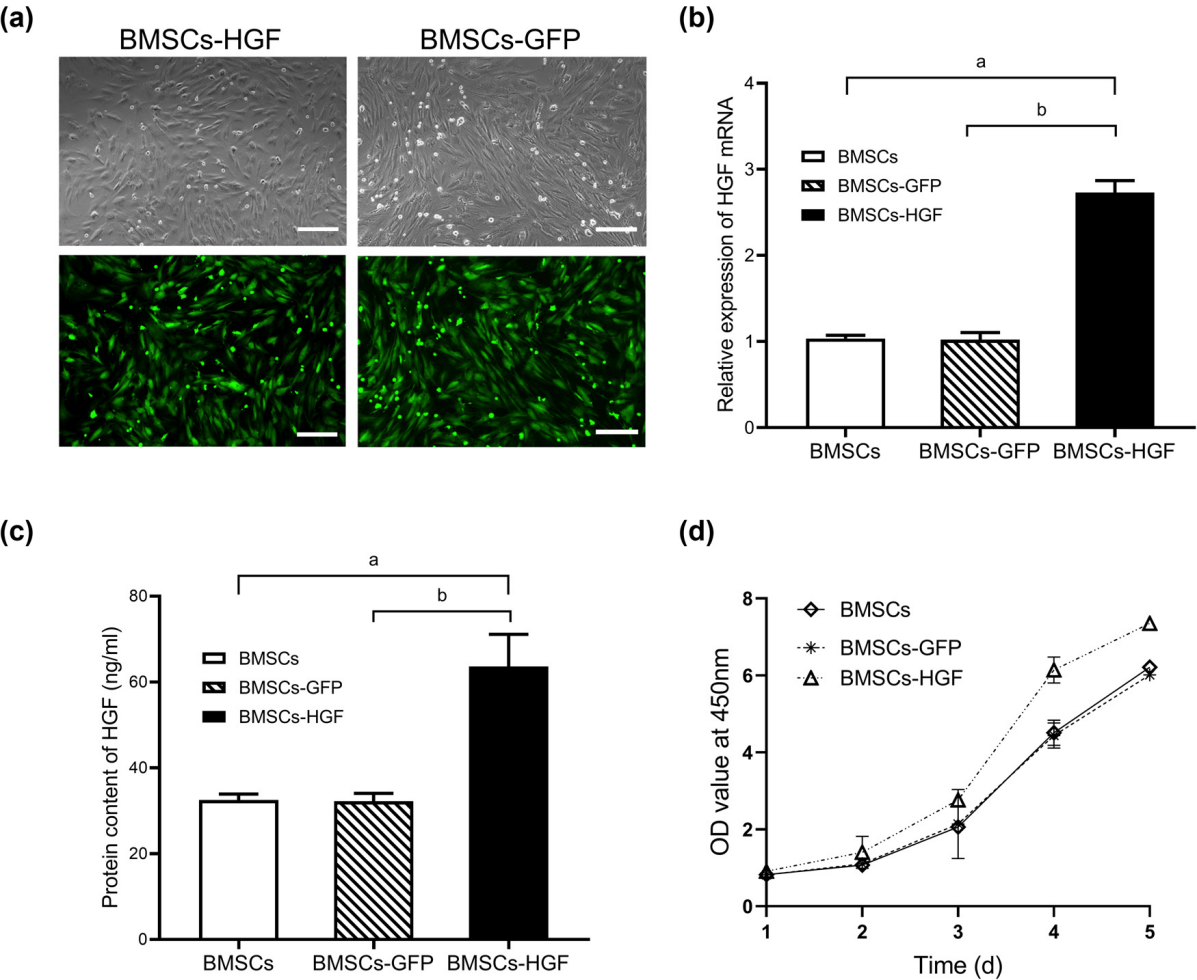
Ad-HGF transfection enhances HGF expression and proliferation in BMSCs. (a) GFP expression in BMSCs transfected with Ad-HGF (BMSCs-HGF) and Ad-GFP (BMSCs-GFP) at MOI = 150 by fluorescence microscopy, respectively. Scale bar = 25 µm. (b) After 48 h of HGF gene transduction, the level of HGF mRNA in BMSCs was determined by RT-qPCR. (c) The protein level of HGF in the cultured BMSC supernatant was measured using ELISA. (d) CCK-8 assays were performed at 1, 2, 3, 4, and 5 days after transfection to determine the proliferation of BMSCs. Ad-HGF transfection promoted the proliferation of BMSCs compared with BMSCs-GFP and nontransfected BMSCs (*P* < 0.05). Ad-GFP transfection did not affect the proliferation of BMSCs (*P* > 0.05). Data are expressed as the mean ± SD. ((a) *P* < 0.001 vs BMSCs; (b) *P* < 0.001 vs BMSCs-GFP).

BMSCs were transfected with Ad-HGF (BMSCs-HGF) and Ad-GFP (BMSCs-GFP), respectively. To optimize the efficiency of adenovirus infection in BMSCs, we calculated the percentage of GFP-positive cells in BMSCs-HGF and BMSCs-GFP at different MOIs. Figure 2a shows that high transfection efficiency and low toxicity could be achieved by Ad-HGF and Ad-GFP at an MOI = 150, at which transduction efficiency was more than 90%. Therefore, BMSCs were transfected with Ad-HGF and Ad-GFP at an MOI of 150 in the subsequent experiments.

Expression of HGF was evaluated by RT-qPCR and ELISA at 48 h after transfection. As shown in Figure 2b, RT-qPCR analysis revealed that the HGF mRNA level was significantly increased in BMSCs-HGF compared with BMSCs-GFP and nontransfected BMSCs, respectively (both *P* < 0.001). Next, we determined the protein content of HGF in the supernatant from the cultured BMSCs by ELISA (Figure 2c). Similar to the results of RT-qPCR, the ELISA results showed that the HGF concentration in BMSCs-HGF was at a higher level than those in BMSCs-GFP and nontransfected BMSCs, respectively. CCK-8 assay was performed to determine the proliferation of BMSCs. As shown in Figure 2d, Ad-HGF promoted the proliferation of BMSCs, compared with that of the nontransfected BMSCs (*P* < 0.05), while Ad-GFP did not affect the HGF expression or proliferation of BMSCs (both *P* > 0.05).

### 3.3 Transplantation of BMSCs-HGF attenuates HIBD in newborn rats

**Figure 3:**
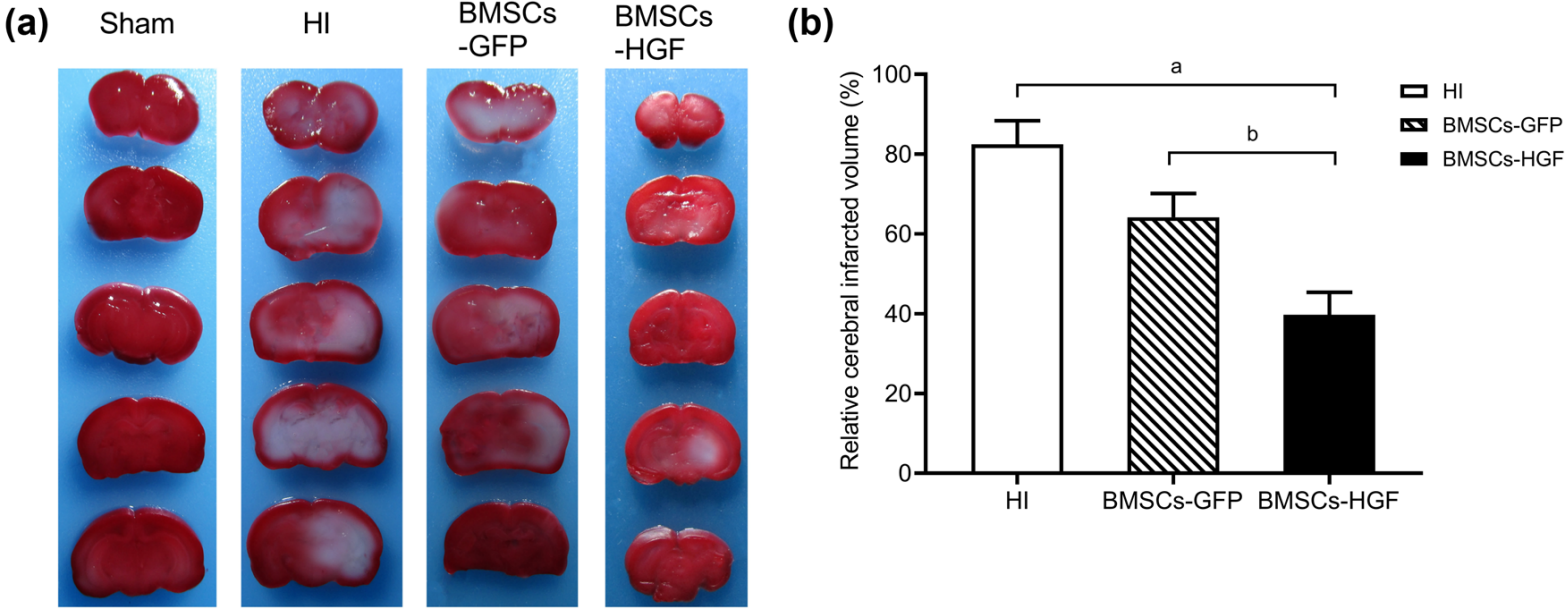
Evaluation of the cerebral infarct volume using TTC staining in different experimental groups. (a) Representative images of TTC-stained brain sections at 72 h after BMSC transplantation. The infarct area is white, while normal tissues are stained in red. (b) Histograms representing the relative quantitative evaluation of the cerebral infarct volume. Data are expressed as the mean ± SD. ((a) *P* < 0.001 vs BMSCs; (b) *P* < 0.001 vs BMSCs-GFP).

To assess the extent of the infarct area at 72 h after HI insult, brains were sliced and stained with TTC. As shown in Figure 3, HI insult induced brain infarction (82.45% ± 5.91%), while the infarct volume was significantly reduced in the BMSCs-GFP and BMSCs-HGF groups, respectively (both *P* < 0.001). Interestingly, an additional, significant reduction of the infarct volume was observed in the BMSCs-HGF group compared with the BMSCs-GFP group (39.70 ± 5.68% vs 64.18 ± 5.98%; *P* < 0.001). These results demonstrate that BMSCs exerted neuroprotective effects and that HGF overexpression increases the ability of BMSCs to protect against HI injury.

### 3.4 HGF expression in the brains of newborn rats submitted to HI

**Figure 4:**
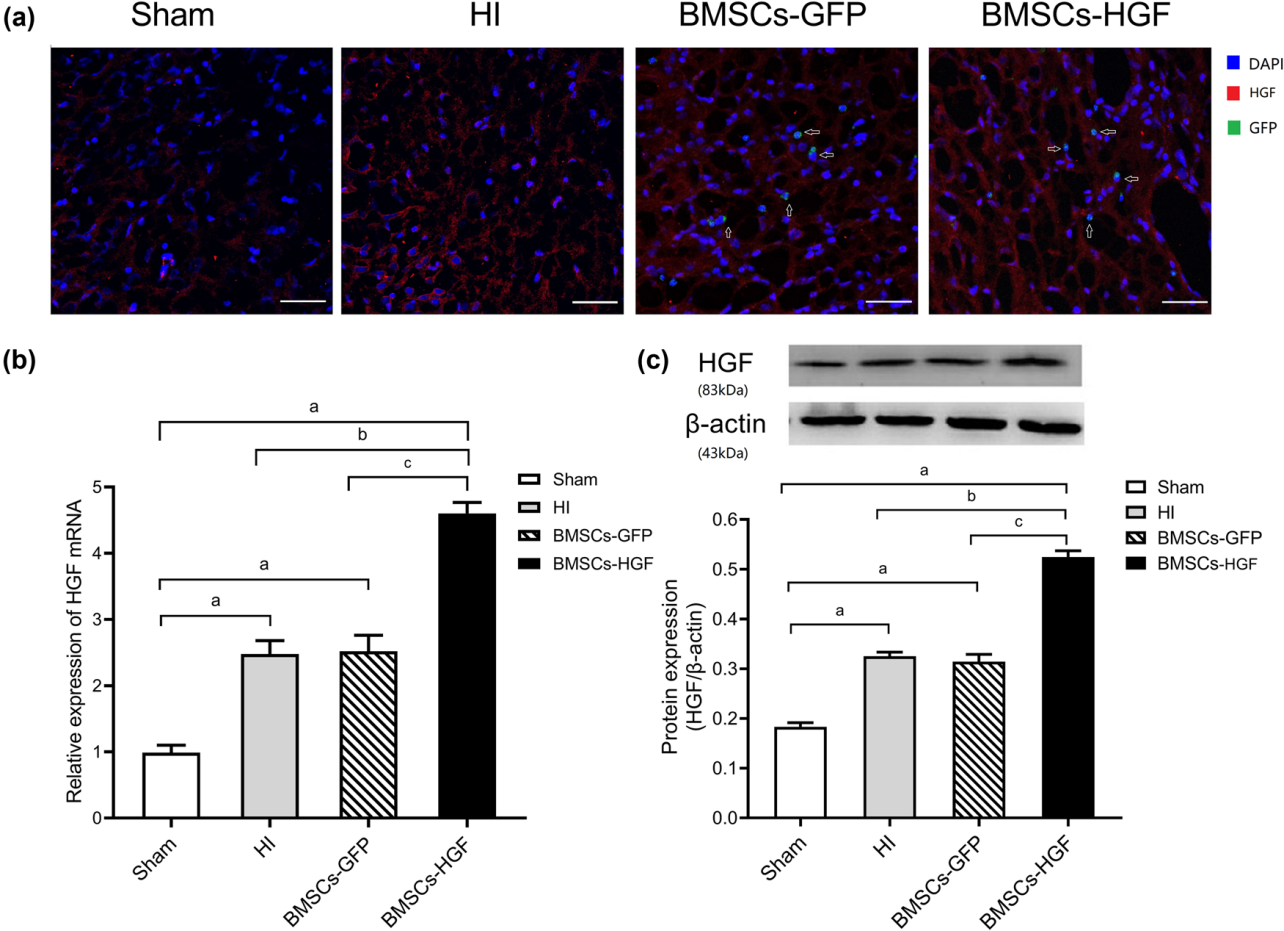
Expression of HGF in different experimental groups after BMSC transplantation. (a) Cerebral expression of HGF determined by immunofluorescence at 24 h after BMSC transplantation. Blue: DAPI; red: HGF; green: GFP (also indicated by arrows). Scale bar = 50 µm. (b) Determination of relative HGF mRNA expression by RT- qPCR. (c) Determination of relative HGF protein expression by western blotting. Data are expressed as the mean ± SD. ((a) *P* < 0.001 vs Sham; (b) *P* < 0.001 vs HI; (c) *P* < 0.001 vs BMSCs-GFP).

At 72 h after the transplantation of BMSCs, HGF expression in the brain was evaluated by immunofluorescence staining. As shown in Figure 4a, the HI insult increased HGF-specific immunofluorescence signals, compared to Sham rats. BMSCs-HGF rats exhibited the strongest red fluorescence, indicating elevated HGF overexpression. Notably, BMSC-specific green fluorescence was detected in the BMSCs-HGF group, indicating the migration of BMSCs-HGF after transplantation. HGF expression in the brain was also quantified by RT-qPCR. Specifically, in HI model rats, a 2.5-fold increase in the level of HGF mRNA was measured at 72 h after the HI insult, compared with that in Sham animals (*P* < 0.001; Figure 4b). This result implies that HI insults were able to induce a slight elevation in HGF expression. However, there was no difference in HGF mRNA expression between the HI model and BMSCs-GFP groups at 72 h after HI insult (*P* > 0.05; Figure 4b), indicating that transplanted BMSCs did not affect the brain HGF expression. HGF mRNA expression was highest in the BMSCs-HGF group and was more than 4-fold higher in the latter group compared with Sham rats (*P* < 0.001; Figure 4b). To further verify the differences in HGF expression, western blotting was used to examine HGF protein levels (Figure 4c) and yielded similar results to the RT-qPCR analysis. Our findings suggest that transplantation of HGF-overexpressing BMSCs is effective and increased HGF expression, mainly secreted by BMSCs-HGF.

### 3.5 Transplantation of BMSCs-HGF modulates the ERK pathway

**Figure 5:**
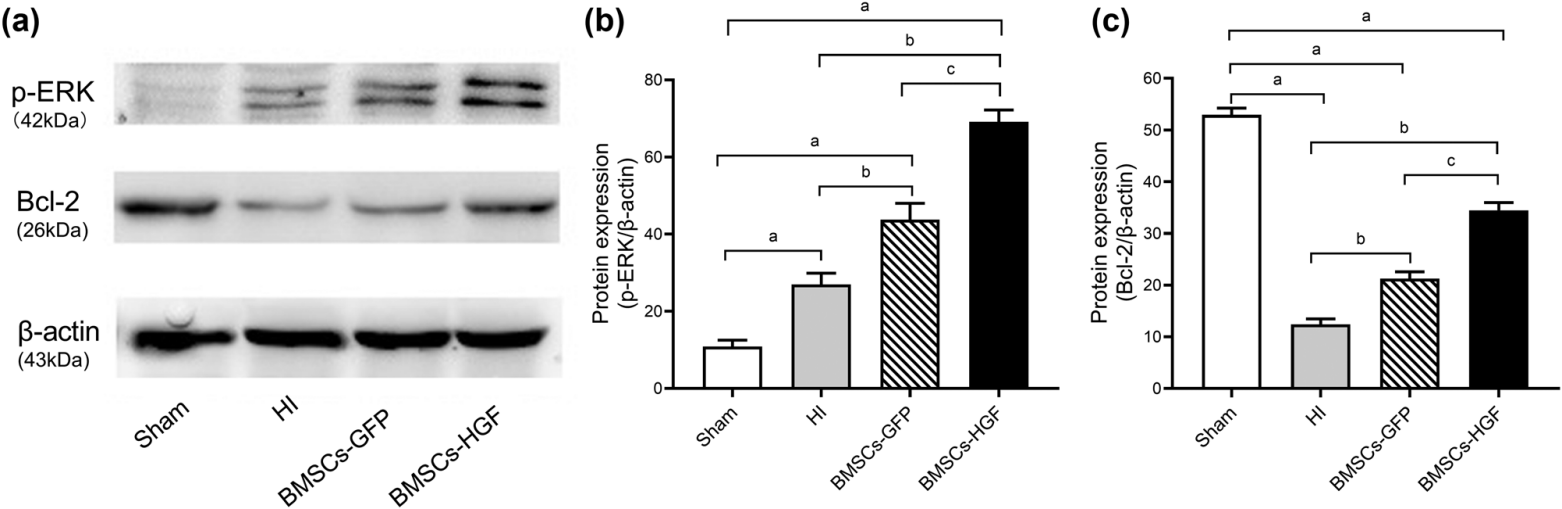
Expression of p-ERK and Bcl-2 in different experimental groups after BMSC transplantation. (a) Representative western blots showing expression of p-ERK and Bcl-2 protein in neonatal rat brains. β-Actin served as the loading control. (b) Histograms representing the relative quantitative evaluation of p-ERK protein. (c) Histograms representing the relative quantitative evaluation of Bcl-2 protein. Data are expressed as the mean ± SD. ((a) *P* < 0.001 vs Sham; (b) *P* < 0.001 vs HI; (c) *P* < 0.001 vs BMSCs-GFP).

To further investigate the mechanisms responsible for BMSCs-HGF-induced neuroprotection, we focused on the downstream ERK pathway that was mediated by HGF signaling. We measured levels of phosphorylated ERK (p-ERK) and of the antiapoptotic protein Bcl-2 in brain tissues in the different experimental groups (Figure 5). We found that p-ERK was slightly upregulated after HI (*P* < 0.001 vs Sham), while Bcl-2 was significantly downregulated (*P* < 0.001 vs Sham). BMSCs-HGF implantation increased levels of p-ERK and Bcl-2, while BMSCs-GFP implantation had comparatively less of an impact on expression levels of these proteins. These results indicate that the ERK signaling pathway is probably involved in the mechanisms underlying the neuroprotective effects of HGF-overexpressing BMSCs.

### 3.6 Inhibition of the ERK pathway abolishes the neuroprotective effect of BMSCs-HGF against OGD

**Figure 6:**
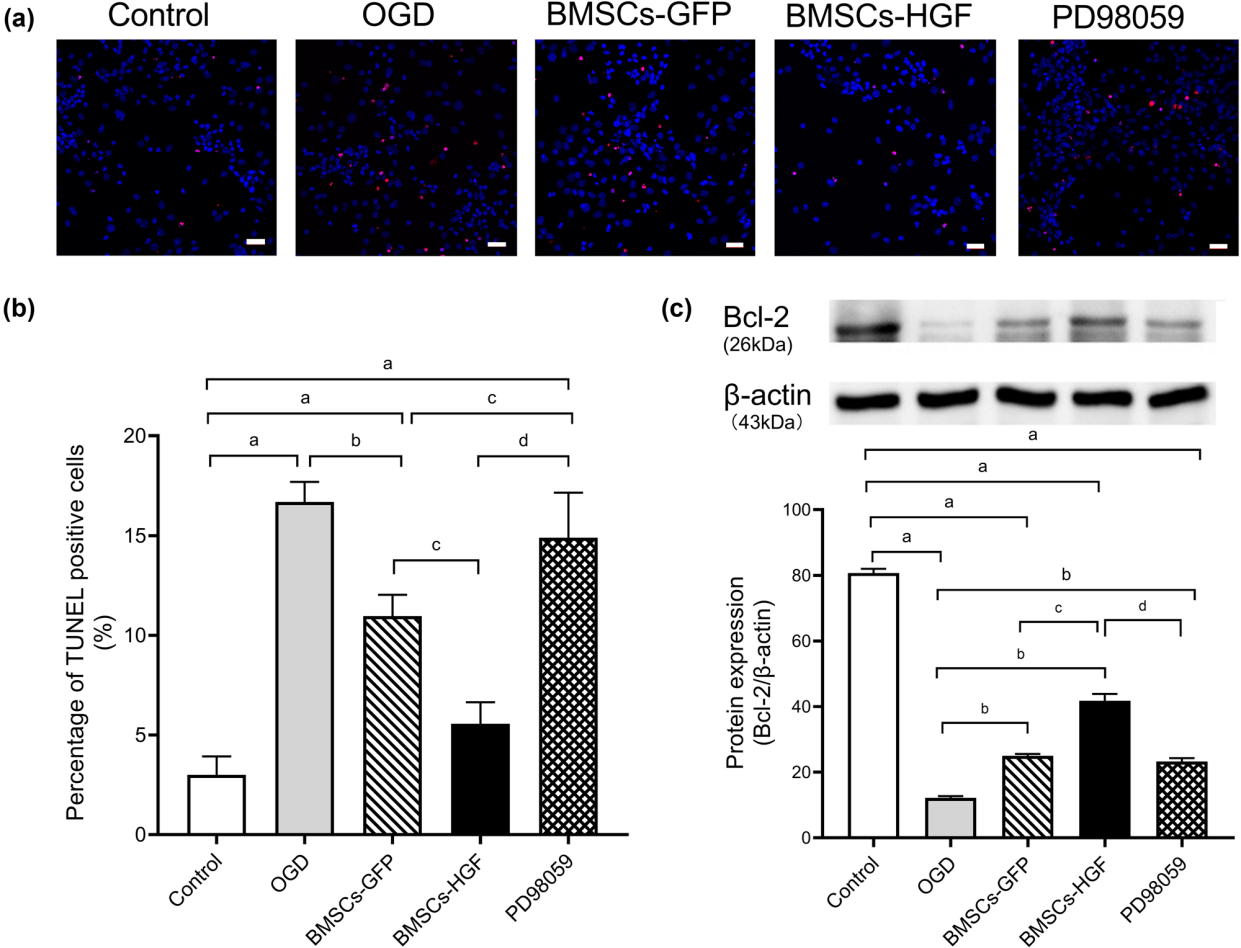
Blocking the phosphorylation of ERK abolishes the effects of BMSCs-HGF on neurons. (a) Apoptosis of neurons induced by OGD in different experimental groups was determined using TUNEL staining. Blue: DAPI; red: TUNEL. Scale bar = 25 µm. (b) Histograms representing the percentage of TUNEL positive neurons by quantitative analysis. (c) Upper panel: representative western blots showing expression of Bcl-2 protein in neurons. Lower panel: histograms representing the relative quantitative evaluation of Bcl-2 protein. Data are expressed as the mean ± SD. ((a): *P* < 0.001 vs Sham; (b) *P* < 0.001 vs OGD; (c) *P* < 0.001 vs BMSCs-GFP; (d) *P* < 0.001 vs BMSCs-HGF).

To further explore the neuroprotective effects and the underlying mechanisms of BMSCs-HGF against HI insults, we cultured BMSCs-HGF with cortical neurons using a co-culture model in a Transwell plate system and simulated the HI insult using OGD. Cell apoptosis was determined using TUNEL staining (Figure 6a and b). The results demonstrated that co-culture with BMSCs decreased the apoptosis induced by OGD. Compared to co-culture with BMSCs-GFP, overexpression of HGF enhanced the protective effects of BMSCs on cortical neurons against OGD. Administration of PD98059, a selective antagonist that inhibits the phosphorylation and activation of ERK, reversed the neuroprotective effect induced by co-culture with BMSCs-HGF.

Similar to the results in the animal models, the western blotting analysis (Figure 6c) showed that Bcl-2 was downregulated in cortical neurons after OGD. However, BMSC transplantation increased Bcl-2 levels in cortical neurons, with overexpression of HGF further enhancing the upregulation of Bcl-2 mediated by BMSCs. Moreover, inhibiting phosphorylation of ERK using PD98059 abolished the upregulation of Bcl-2 induced by BMSCs-HGF (Figure 6c). Collectively, these data suggest that overexpression of HGF in BMSCs regulates Bcl-2 expression via ERK phosphorylation.

## 4 Discussion

In this study, neuroprotective effects of gene-modified BMSCs in neonatal HIBD were investigated. The HGF gene was transferred into BMSCs by adenoviral vectors to conduct HGF-modified BMSCs. The HGF gene was stably expressed in BMSCs-HGF with higher levels of mRNA and protein than those in nontransfected BMSCs. Then, BMSCs-HGF were administered intraventricularly in a neonatal HIBD rat model. We found that transplantation of BMSCs-HGF significantly elevated HGF levels in the recipient's brains, and there was no difference in HGF expression between the BMSCs-GFP group and HI group. These results suggested HGF-modified BMSCs could sustain HGF overexpressing after transplantation and BMSC itself did not increase HGF expression *in vivo*. However, further investigations are still needed to explore the underlying mechanisms of the increased HGF secretion in brain tissues after transplantation of HGF-modified BMSCs, such as “Where did the increased HGF come from? From HGF-modified BMSCs directly? Or from other types of cells in the recipient brain modulated by HGF-modified BMSCs?”.

Notably, HGF levels in brain tissues of the HI group were slightly increased after HIBD. We speculated that HI insult activated the endogenous HGF secretion in the brain for self-repair. Compared with BMSCs-GFP, BMSCs-HGF significantly decreased infarct volumes in recipient's brains, indicating overexpressing HGF by HGF-gene modification enhanced the neuroprotective effects of BMSCs. Similar results, BMSCs decreased neuronal apoptosis induced by OGD and HGF overexpressing amplified the neuroprotective effects of BMSCs, were also found in an *in vitro* study.

HGF and its receptor, c-Met, are widely expressed in the central nervous system and play potent neuroprotective effects. Shimamura et al. found HGF alleviated brain ischemic injury without exacerbating cerebral edema through promoting angiogenesis and inhibiting neuronal apoptosis [23]. Moreover, HGF could promote neural stem cell proliferation and self-renewal, and HGF could enhance neurogenesis by stimulating the proliferation of neural precursor cells and protecting them against hypoxic injury [29,30].

As a pleiotropic growth factor, HGF exerts mitogenic and anti-apoptotic properties through binding to the c-Met receptor and activating multiple downstream signaling pathways, including ERK/Bcl-2 signaling pathway [31]. After binding with HGF, two tyrosine residues (Y1234 and Y1235) in c-Met undergo autophosphorylation and their intrinsic kinase activities are activated. This leads to the phosphorylation of Y1349 and Y1356 of c-Met, ERK phosphorylation in tyrosine, and activation of the ERK pathway [32,33]. ERK pathway is widely involved in cell proliferation, differentiation, migration, and apoptosis [34]. ERK phosphorylation upregulates Bcl-2 [35]. Bcl-2 is a proto-oncogene that belongs to a family of apoptosis-modulating proteins and plays an important anti-apoptotic function. It has been associated with inhibiting apoptosis by inhibiting cytochrome C release from the mitochondria, regulating calcium flux, and maintaining the integrity of the mitochondrial membrane [36–39].

In the present study, using western blots to detect ERK activation, we found that BMSCs-HGF transplantation markedly increased the level of p-ERK, consistent with an increased level of Bcl-2. Suppression of ERK phosphorylation using a specific inhibitor, PD98059, reversed the upregulation of Bcl-2 and the protective effects against HI. Overall, the results of this study revealed that BMSCs-HGF-mediated ERK/Bcl-2 pathway modulation correlated with anti-apoptotic effects on neurons.

It is reported that more than 20% of survivors present with long-term neurological disabilities, including cerebral palsy, epilepsy, and mental retardation [40]. However, we mainly focused on the protective effects of BMSCs-HGF on neuronal injury; long-term neurological outcomes with BMSCs-HGF transplantation were not evaluated in the current study. Moreover, the actual mechanisms underlying HIBD relate to multiple factors. For example, after HIBD, the ultrastructure of the blood–brain barrier changes, leading to the development of brain edema [41]. Doeppner et al. found that HGF stabilized the integrity of the BBB by inactivating matrix metalloproteases [30]. All of the findings indicate that HGF is a potential candidate for HIBD treatment. However, it is still necessary to identify the appropriate administration methods, dose, and timing, and address the risk of tumorigenesis; however, gene therapy may solve the issue of HGF degradation. Using BMSCs as the host cells for gene transfer is not only safer than using viral vectors but also offers the advantages of cell transplantation, enhancing the therapeutic effects on HIBD. Importantly, further studies are still needed to explore the protective effects of BMSCs-HGF on HIBD more thoroughly, including the potential side effects and the impacts on long-term neurological outcomes. These investigations will provide more clues and evidence regarding the efficacy and safety of BMSCs-HGF for therapeutic applications in the clinic.

## 5 Conclusion

In summary, our findings indicate that transfection with Ad-HGF upregulates HGF expression and cell proliferation in BMSCs and that transplantation of BMSCs overexpressing HGF protects the HIBD model neonatal rats against neuronal damage. Moreover, modulation of the ERK/Bcl-2 signaling pathway by HGF might be the mechanism underlying this neuroprotective effect. However, further studies are required to explore the best way to address several issues, including the mode of administration, the risk of oncogenesis, and the impacts on long-term prognosis and on other organs.

## References

[1] Douglas-Escobar M, Weiss MD. Hypoxic-ischemic encephalopathy: a review for the clinician. JAMA Pediatr. 2015;169:397–403. 10.1001/jamapediatrics.2014.3269 25685948

[2] Finder M, Boylan GB, Twomey D, Ahearne C, Murray DM, Hallberg B. Two-year neurodevelopmental outcomes after mild hypoxic ischemic encephalopathy in the era of therapeutic hypothermia. JAMA Pediatr. 2020;174:48–55. 10.1001/jamapediatrics.2019.4011 PMC6865301 31710357

[3] Lodygensky GA, Battin MR, Gunn AJ. Mild neonatal encephalopathy – how, when, and how much to treat? JAMA Pediatr. 2018;172:3–4. 10.1001/jamapediatrics.2017.3044 29114743

[4] Tagin MA, Woolcott CG, Vincer MJ, Whyte RK, Stinson DA. Hypothermia for neonatal hypoxic ischemic encephalopathy: an updated systematic review and meta-analysis. Arch Pediatri Adolesc Med. 2012;166:558–66. 10.1001/archpediatrics.2011.1772 22312166

[5] Qin X, Cheng J, Zhong Y, Mahgoub OK, Akter F, Fan Y, et al. Mechanism and treatment related to oxidative stress in neonatal hypoxic-ischemic encephalopathy. Front Mol Neurosci. 2019;12:88. 10.3389/fnmol.2019.00088 PMC6470360 31031592

[6] Li B, Concepcion K, Meng X, Zhang L. Brain-immune interactions in perinatal hypoxic-ischemic brain injury. Prog Neurobiol. 2017;159:50–68. 10.1016/j.pneurobio.2017.10.006 PMC5831511 29111451

[7] Serrenho I, Rosado M, Dinis A, M Cardoso C, Grãos M, Manadas B, et al. Stem cell therapy for neonatal hypoxic-ischemic encephalopathy: a systematic review of preclinical studies. Int J Mol Sci. 2021;22:3142. 10.3390/ijms22063142 PMC8003344 33808671

[8] Charbord P. Bone marrow mesenchymal stem cells: historical overview and concepts. Hum Gene Ther. 2010;21:1045–56. 10.1089/hum.2010.115 PMC4823383 20565251

[9] Xu L, Liu Y, Sun Y, Wang B, Xiong Y, Lin W, et al. Tissue source determines the differentiation potentials of mesenchymal stem cells: a comparative study of human mesenchymal stem cells from bone marrow and adipose tissue. Stem Cell Res Ther. 2017;8:275. 10.1186/s13287-017-0716-x PMC5718061 29208029

[10] Wei L, Fraser JL, Lu ZY, Hu X, Yu SP. Transplantation of hypoxia preconditioned bone marrow mesenchymal stem cells enhances angiogenesis and neurogenesis after cerebral ischemia in rats. Neurobiol Dis. 2012;46:635–45. 10.1016/j.nbd.2012.03.002 PMC3353023 22426403

[11] Cong XQ, Li Y, Zhao X, Dai YJ, Liu Y. Short-term effect of autologous bone marrow stem cells to treat acute myocardial infarction: a meta-analysis of randomized controlled clinical trials. J Cardiovasc Transl Res. 2015;8:221–31. 10.1007/s12265-015-9621-9 25953677

[12] Shichinohe H, Kawabori M, Iijima H, Teramoto T, Abumiya T, Nakayama N, et al. Research on advanced intervention using novel bone marrOW stem cell (RAINBOW): a study protocol for a phase I, open-label, uncontrolled, dose-response trial of autologous bone marrow stromal cell transplantation in patients with acute ischemic stroke. BMC Neurol. 2017;17:179. 10.1186/s12883-017-0955-6 PMC5591569 28886699

[13] Tadokoro K, Fukui Y, Yamashita T, Liu X, Tsunoda K, Shang J, et al. Bone marrow stromal cell transplantation drives molecular switch from autophagy to the ubiquitin-proteasome system in ischemic stroke mice. J Stroke Cerebrovasc Dis. 2020;29:104743. 10.1016/j.jstrokecerebrovasdis.2020.104743 32127256

[14] Song M, Mohamad O, Gu X, Wei L, Yu SP. Restoration of intracortical and thalamocortical circuits after transplantation of bone marrow mesenchymal stem cells into the ischemic brain of mice. Cell Transplant. 2013;22:2001–15. 10.3727/096368912X657909 23069268

[15] Li L, Chu L, Ren C, Wang J, Sun S, Li T, et al. Enhanced migration of bone marrow-derived mesenchymal stem cells with tetramethylpyrazine and its synergistic effect on angiogenesis and neurogenesis after cerebral ischemia in rats. Stem Cells Dev. 2019;28:871–81. 10.1089/scd.2018.0254 31038013

[16] Pirzad Jahromi G, Shabanzadeh Pirsaraei A, Sadr SS, Kaka G, Jafari M, Seidi S, et al. Multipotent bone marrow stromal cell therapy promotes endogenous cell proliferation following ischemic stroke. Clin Exp Pharmacol Physiol. 2015;42:1158–67. 10.1111/1440-1681.12466 26218989

[17] Yoo SW, Kim SS, Lee SY, Lee HS, Kim HS, Lee YD, et al. Mesenchymal stem cells promote proliferation of endogenous neural stem cells and survival of newborn cells in a rat stroke model. Exp Mol Med. 2008;40:387–97. 10.3858/emm.2008.40.4.387 PMC2679267 18779651

[18] Cortez-Toledo E, Rose M, Agu E, Dahlenburg H, Yao W, Nolta JA, et al. Enhancing retention of human bone marrow mesenchymal stem cells with prosurvival factors promotes angiogenesis in a mouse model of limb ischemia. Stem Cells Dev. 2019;28:114–9. 10.1089/scd.2018.0090 PMC6350414 30398391

[19] Samakova A, Gazova A, Sabova N, Valaskova S, Jurikova M, Kyselovic J. The PI3k/Akt pathway is associated with angiogenesis, oxidative stress and survival of mesenchymal stem cells in pathophysiologic condition in ischemia. Physiol Res. 2019;68:S131–8. 10.33549/physiolres.934345 31842576

[20] Kachgal S, Putnam AJ. Mesenchymal stem cells from adipose and bone marrow promote angiogenesis via distinct cytokine and protease expression mechanisms. Angiogenesis. 2011;14:47–59. 10.1007/s10456-010-9194-9 PMC3369878 21104120

[21] He J, Wang C, Sun Y, Lu B, Cui J, Dong N, et al. Exendin-4 protects bone marrow-derived mesenchymal stem cells against oxygen/glucose and serum deprivation-induced apoptosis through the activation of the cAMP/PKA signaling pathway and the attenuation of ER stress. Int J Mol Med. 2016;37:889–900. 10.3892/ijmm.2016.2509 PMC4790651 26935620

[22] Nakamura T, Sakai K, Nakamura T, Matsumoto K. Hepatocyte growth factor twenty years on: much more than a growth factor. J Gastroenterol Hepatol. 2011;26:188–202. 10.1111/j.1440-1746.2010.06549.x 21199531

[23] Shimamura M, Sato N, Oshima K, Matsumoto K. Novel therapeutic strategy to treat brain ischemia: overexpression of hepatocyte growth factor gene reduced ischemic injury without cerebral edema in rat model. Circulation. 2004;109:424–31. 10.1161/01.CIR.0000109496.82683.49 14707023

[24] Zeng W, Ju R, Mao M. Therapeutic potential of hepatocyte growth factor against cerebral ischemia (Review). Exp Ther Med. 2015;9:283–8. 10.3892/etm.2014.2133 PMC4280917 25574187

[25] Lotfy A, Salama M, Zahran F, Jones E, Badawy A, Sobh M. Characterization of mesenchymal stem cells derived from rat bone marrow and adipose tissue: a comparative study. Int J Stem Cells. 2014;7:135–42. 10.15283/ijsc.2014.7.2.135 PMC4249896 25473451

[26] Vannucci RC, Vannucci SJ. Perinatal hypoxic-ischemic brain damage: evolution of an animal model. Dev Neurosci. 2005;27:81–6. 10.1159/000085978 16046840

[27] Zhang X, Zhang Q, Li W, Nie D, Chen W, Xu C, et al. Therapeutic effect of human umbilical cord mesenchymal stem cells on neonatal rat hypoxic–ischemic encephalopathy. J Neurosci Res. 2014;92:35–45. 10.1002/jnr.23304 24265136

[28] Robert F, Cloix JF, Hevor T. Ultrastructural characterization of rat neurons in primary culture. Neuroscience. 2012;200:248–60. 10.1016/j.neuroscience.2011.10.002 22079571

[29] Nicoleau C, Benzakour O, Agasse F, Thiriet N, Petit J, Prestoz L, et al. Endogenous hepatocyte growth factor is a niche signal for subventricular zone neural stem cell amplification and self-renewal. Stem Cells. 2009;27:408–19. 10.1634/stemcells.2008-0226 18988709

[30] Doeppner TR, Kaltwasser B, ElAli A, Zechariah A, Hermann DM, Bähr M. Acute hepatocyte growth factor treatment induces long-term neuroprotection and stroke recovery via mechanisms involving neural precursor cell proliferation and differentiation. J Cereb Blood Flow Metab. 2011;31:1251–62. 10.1038/jcbfm.2010.211 PMC3099629 21119693

[31] Matsumoto K, Nakamura T. Hepatocyte growth factor: molecular structure, roles in liver regeneration, and other biological functions. Crit Rev Oncog. 1992;3:27–54. 1312869

[32] Noriega-Guerra H, Freitas VM. Extracellular matrix influencing HGF/c-MET signaling pathway: impact on cancer progression. Int J Mol Sci. 2018;19:3300. 10.3390/ijms19113300 PMC6274944 30352967

[33] Organ SL, Tsao MS. An overview of the c-MET signaling pathway. Ther Adv Med Oncol. 2011;3:S7–19. 10.1177/1758834011422556 PMC3225017 22128289

[34] Lu N, Malemud CJ. Extracellular signal-regulated kinase: a regulator of cell growth, inflammation, chondrocyte and bone cell receptor-mediated gene expression. Int J Mol Sci. 2019;20:3792. 10.3390/ijms20153792 PMC6696446 31382554

[35] Tamura Y, Simizu S, Osada H. The phosphorylation status and anti-apoptotic activity of Bcl-2 are regulated by ERK and protein phosphatase 2A on the mitochondria. FEBS Lett. 2004;569:249–55. 10.1016/j.febslet.2004.06.003 15225643

[36] Marsden VS, Ekert PG, Van Delft M, Vaux DL, Adams JM, Strasser A. Bcl-2-regulated apoptosis and cytochrome c release can occur independently of both caspase-2 and caspase-9. J Cell Biol. 2004;165:775–80. 10.1083/jcb.200312030 PMC2172407 15210727

[37] Thangarajan S, Vedagiri A, Somasundaram S, Sakthimanogaran R, Murugesan M. Neuroprotective effect of morin on lead acetate- induced apoptosis by preventing cytochrome c translocation via regulation of Bax/Bcl-2 ratio. Neurotoxicol Teratol. 2018;66:35–45. 10.1016/j.ntt.2018.01.006 29353014

[38] Lam M, Dubyak G, Chen L, Nuñez G, Miesfeld RL, Distelhorst CW. Evidence that BCL-2 represses apoptosis by regulating endoplasmic reticulum-associated Ca2+ fluxes. Proc Natl Acad Sci USA. 1994;91:6569–73. 10.1073/pnas.91.14.6569 PMC44244 8022822

[39] Lewis A, Hayashi T, Su TP, Betenbaugh MJ. Bcl-2 family in inter-organelle modulation of calcium signaling; roles in bioenergetics and cell survival. J Bioenerg Biomembr. 2014;46:1–15. 10.1007/s10863-013-9527-7 PMC4529064 24078116

[40] Kharoshankaya L, Stevenson NJ, Livingstone V, Murray DM, Murphy BP, Ahearne CE, et al. Seizure burden and neurodevelopmental outcome in neonates with hypoxic–ischemic encephalopathy. Dev Med Child Neurol. 2016;58:1242–48. 10.1111/dmcn.13215 PMC5214689 27595841

[41] Ballabh P, Braun A, Nedergaard M. The blood-brain barrier: an overview: structure, regulation, and clinical implications. Neurobiol Dis. 2004;16:1–13. 10.1016/j.nbd.2003.12.016 15207256

